# Food-Grade Bigels with Potential to Replace Saturated and Trans Fats in Cookies

**DOI:** 10.3390/gels8070445

**Published:** 2022-07-17

**Authors:** Marcela Quilaqueo, Nicole Iturra, Ingrid Contardo, Sonia Millao, Eduardo Morales, Mónica Rubilar

**Affiliations:** 1 Department of Chemical Engineering, Faculty of Engineering and Science, Universidad de La Frontera, Francisco Salazar 01145, Temuco 4811230, Chile; n.iturra03@ufromail.cl (N.I.); sonia.millao@gmail.com (S.M.); 2 Scientific and Technological Bioresource Nucleus, BIOREN, Universidad de La Frontera, Avenida Francisco Salazar 01145, Temuco 4811230, Chile; eduardo.morales@ufrontera.cl; 3 Biopolymer Research & Engineering Laboratory (BiopREL), School of Nutrition and Dietetics, Faculty of Medicine, Universidad de los Andes, Monseñor Álvaro del Portillo 12.455, Las Condes 7550000, Chile; icontardo@uandes.cl; 4 Centro de Investigación e Innovación Biomédica (CiiB), Universidad de los Andes, Monseñor Álvaro del Portillo 12.455, Las Condes 7550000, Chile

**Keywords:** canola oil, beeswax, sodium alginate, carboxymethylcellulose, hydrogel, oleogel, bigel

## Abstract

Fats play multiple roles in determining the desirable characteristics of foods. However, there are health concerns about saturated and trans fats. Bigels have been proposed as a novel fat replacer in foods. This research evaluated the role of the type of hydrogel in the development of bigels to be used as fat replacers in cookies. Bigels were made with beeswax/canola oil oleogel and sodium alginate and carboxymethylcellulose hydrogels. The results showed that the peroxide value and binding capacity of bigels were affected by the type of hydrogel used. However, their fatty acid profile, *p*-anisidine value, oxidative stability, and texture remained unchanged. Using bigels as fat replacers, cookies were obtained with a hardness similar to those with original shortening, showing the potential of bigels for use in foods.

## 1. Introduction

Fats and oils are important raw materials for several food products. Solid fat owes much of its functionality and properties to saturated fatty acids (SFAs) and trans fatty acids (TFAs) because these fatty acids, present in the form of triacylglycerols, result in the assembly of a colloidal or supracolloidal particle network, responsible for structuring the fat into a solid or solid-like material [1]. However, there are health concerns about solid fats, that is, SFAs and TFAs. The consumption of SFAs instead of polyunsaturated fatty acids (PUFAs) is related to a rise in low-density lipoprotein cholesterol levels. Although a debate remains regarding the negative effects of SFAs on health, if we accept the premise that consumers demand foods free of SFAs, then there is a pressing need for alternative oil structurants [1,2]. TFAs, a subclass of unsaturated fatty acids, are especially harmful due to the fact their intake is related to raising the level of low-density lipoprotein cholesterol and to high-density lipoprotein reduction [2].

Nowadays, the options to remove the SFAs and TFAs maintaining the desirable physical properties of foods are limited. In this regard, the bigel (also called hybrid gel) is a novel solid-like formulation produced from a combination of an oleogel and a hydrogel at a high shear rate [3]. For their part, oleogels are soft matter structures that can trap oils in a thermo-reversible gel network, resulting in products with properties similar to solids. Oleogels have great potential applications in food as an alternative to SFAs and TFAs [4,5]. Oleogels produced with beeswax (BW) have been studied with good results; the properties of BW oleogels can be tailored to specific applications by operating with particular process conditions. The functionality of food products containing BW oleogels has been positively evaluated as a total or partial fat replacer, for example, in cookies [6,7].

On the other hand, hydrogels are three-dimensional solid networks formed by physically or chemically cross-linked hydrophilic polymeric structures that can entangle water or other biological liquid inside their network. Hydrogels present interesting properties such as easy spreadability and miscibility, and their compatibility with a wide range of excipients (i.e., solvents) allows for their use in a wide range of applications [3]. Some non-digestible carbohydrates, such as sodium alginate (ALG) and carboxymethylcellulose (CMC), have shown the potential to improve the quality of low-fat foods [8,9].

Given that bigels are structured by two phases with different polarities (aqueous and oily), they possess the characteristics of both phases; these systems can present better properties than either of the single gels, in terms of the delivery of agents, cooling and moisturizing effects, spreadability, the permeability of drugs through the skin, and stability at room temperature, among others. Currently, bigels are mainly used for pharmaceutical and cosmetic purposes, but minor applications have already been found in the food area [10]. Some food-grade bigels have been studied showing that it is possible to tailor their mechanical properties, rheology, and thermal stability, directing their characteristics to allow for their use in food products as a fat replacer [3,10,11]. The potential of bigels as a fat replacer was shown for the first time in foods by Ghiasi et al. [12], who found that low-fat beef burgers using bigels presented better cooking properties compared to animal fat burgers. Moreover, no significant differences were detected by sensory panelists between control and low-fat burgers in terms of overall acceptability.

Cookies are popular food products composed of flour, sugar, fat, and mainly water. Cookies are high in fat, generally among 20–30% or more on a flour weight basis, which is not desirable from the health point of view and makes them susceptible to oxidative damage [13]. Fat contributes to a desirable mouthfeel and texture and improves flavor perception [14], imparts aeration and stability, and positively contributes to the structure and geometry of cookies [4]. Therefore, fat replacement in cookies could result in a healthier product with prolonged shelf life but with greater hardness and brittleness, and lower crumbliness, than the cookies made with full fat. A great effort has been made to reduce the fat content of cookies while maintaining their sensory properties [13]. For this reason, the authors decided to create low-fat cookies using bigels as a fat replacer. 

The aim of this research was to evaluate the role of the type of hydrogel in the development of bigels to be used as SFAs and TFAs replacers in cookies. ALG and CMC were used as gelling agents to prepare the hydrogels. Bigels were prepared with beeswax/canola oil-based oleogel. Canola oil was chosen to create the oleogels because it is recognized as an oil high in healthier monounsaturated fatty acids (MUFAs) and PUFAs compared to other vegetable oils [4].

## 2. Results and Discussion

### 2.1. Characterization of Bigels

#### 2.1.1. Fatty Acid Profile

Most of the fatty acids found in the canola oil were maintained in BW-O and, in turn, were maintained in the bigels (Table 1). Moreover, trans fatty acids were not detected. The main fatty acids identified in canola oil were palmitic acid (C16), oleic acid (C18:1), linoleic acid (C18:2n6), and α-linolenic acid (C18:3n3), which are consistent with reports by other authors for canola oil [15]. These main fatty acids were maintained through the oleogelation process since BW-O are present in amounts very similar to canola oil. It can be observed that in the oleogel, the palmitic acid and other fatty acids (present in minor amounts) slightly increased their amount, compared to canola oil. This could be due to the BW being mainly a heterogeneous mixture of long-chain esters, n-alkanes, and fatty acids, where the amount of free fatty acids can be 9–11%, as pointed out by some authors [6,16]. Thus, BW can contribute to the fatty acid content of oleogels.

The 4 main fatty acids found in canola oil and BW-O were also found in BW-ALG and BW-CMC bigels, but their concentrations were reduced approximately by half due to bigels being composed of a 50/50 oleogel/hydrogel ratio. 

Canola oil is recognized for its low content of SFAs, high content of MUFA, and low content of erucic acid [17], which is in accordance with what was found in our research. Canola oil had approximately 7.5% of SFAs, 62.29% of MUFAs, and 30.21% of PUFAs, while BW-O had 10.20% of SFAs, 61.81% of MUFAs, and 28.01% of PUFAs. The BW-ALG contained 10.60% SFAs, 62.64% of MUFAs, and 26.75% of PUFAs, which was not very different from the BW-O. Similarly, the BW-CMC bigel presented 10.94% of SFAs, 62.18% of MUFAs, and 26.90% of PUFAs. The results indicate that the structuration of canola oil in bigel matrices containing ALG or CMC as gelling agents allows for the maintenance of the healthy fatty acid profile of the original oil.

#### 2.1.2. Lipid Oxidation and Oxidative Stability 

The *PV* of BW-CMC bigel was significantly lower than that of BW-ALG bigel (Table 2). The *PV* value of the bigels was compared with the *PV* value of an emulsion prepared at the same conditions as the bigels (temperature, mixing speed, and oleogel ratio), but distilled water (without polymer) was used to prepare the sample instead of hydrogel (BW-W); BW-W also had a low *PV*, similar to the BW-CMC bigel. These results suggest that the use of hydrogel in the aqueous phase instead of just water had no advantage of protection against primary oxidation in the bigel preparation process. The *PV* values of bigels were lower than those of the BW-O; this result was expected because bigels contain approximately half of the total lipid content (bigel and oleogel were compared in terms of sample weight). However, the *PV* values of all the analyzed samples (Table 2) are below the maximum allowed by international standards of up to 15 meq O_2_/kg oil [18]. This indicates that the process conditions of the bigel preparation did not promote primary oxidation.

The *AV* values of bigels were similar but significantly lower than that of BW-W (Table 2), suggesting the use of hydrogels could protect the oil against secondary oxidation in the bigel elaboration process. The *AV* is a measure of non-volatile secondary oxidation products, principally aldehydes, and ketones, in oils [19]. Our results indicate that the bigel preparation process did not promote the secondary oxidation of the oil. 

The type of hydrogel used to prepare the bigels did not present a significant difference in the oxidative stability represented by the induction time (Table 2). However, the use of ALG and CMC hydrogels instead of water allows for the improvement of the oxidative stability, evidenced by the significantly lower induction time of BW-W. Many initiators of lipid oxidation reactions are water-soluble, which has important implications because they must either travel through or interact across the interfacial membrane to come into contact with the oil [20]. Moreover, water causes the hydrolysis of triglycerides into di- and mono-glycerides, free fatty acids, and glycerol, which can accelerate oxidation and decomposition [21]. However, when a solute molecule is introduced into pure water, the normal structure organization and interactions with the water molecules are altered. Gelling agents, such as ALG and CMC, increase the viscosity of aqueous solutions and improve emulsion stability by reducing the rate at which particulate matter moves [20]. Thus, the process conditions do not negatively affect the oxidative stability of bigels, and the use of hydrogels presents an advantage over the use of water in the aqueous phase, contributing to the improvement of the oxidative stability of the matrix.

The oxidative stability of bigels showed no significant differences with canola oil. However, the highest oxidative stability was found in the BW-O with the longest induction time. A protective effect of oleogel structurants against oil oxidation has been previously reported; the movement of liquid oil trapped in the gel matrix is hindered by its high viscosity or semi-solid state, thus retarding oxidation [22,23].

#### 2.1.3. X-ray Diffraction, Microstructure, and Binding Capacity

**Figure 1:**
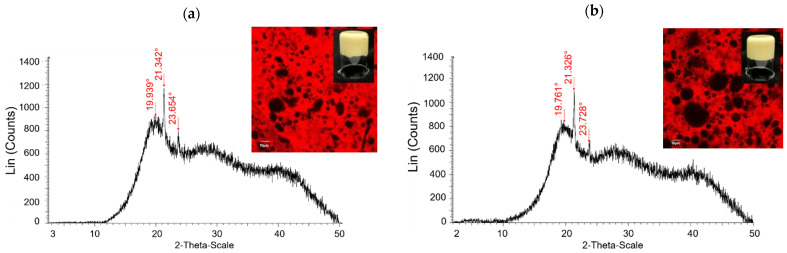

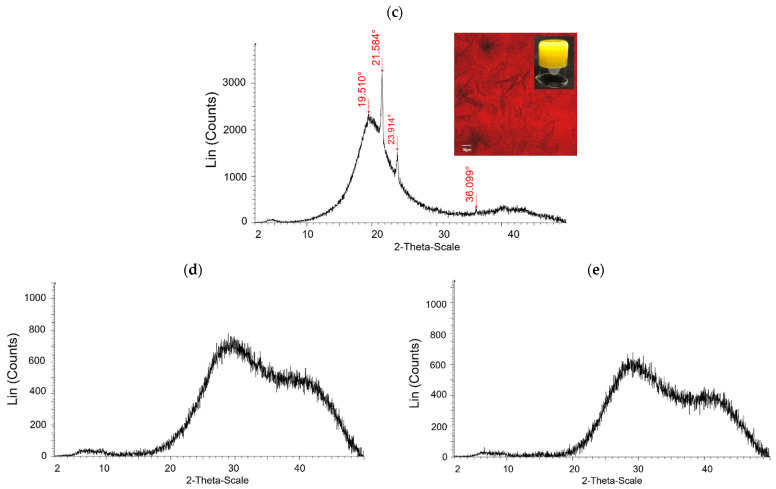
X-ray patterns, confocal images, and structural behavior for (**a**) BW-ALG bigel, (**b**) BW-CMC bigel, (**c**) oleogel, (**d**) hydrogel made from sodium alginate, and (**e**) hydrogel made from carboxymethylcellulose.

The diffraction pattern obtained from X-ray analysis showed that the resulting bigels were a semi-crystalline solid that presented a crystalline and an amorphous component (Figure 1a,b). The crystalline component presented a low degree of crystallinity, while the amorphous component contributed largely to the diffraction pattern. The strong diffraction peaks at 19°, 21°, and 23° (4.5, 4.1, and 3.7 Å, respectively) of the bigels were due to the presence of BW-O. BW-O was a semi-crystalline solid with a high degree of crystallinity (Figure 1c); it showed strong diffraction peaks at 19°, 21°, and 23°, corresponding to d spacing of 4.5, 4.1, and 3.7 Å, respectively. These peaks are characteristics of BW and indicate the presence of β, α, and β’ crystals in the structure [24]. On the other hand, ALG and CMC hydrogels were entirely amorphous (Figure 1d,e); they exhibited broad peaks around 30°–40°. This confirms that the crystalline structure was due to the oleogel, given in turn by BW; however, the presence of hydrogel particles led to the prevalence of the amorphous character of bigels similar to those found by Fasolin, Martins, Cerqueira, and Vicente [11]. Overall, the X-ray diffraction patterns of the BW-ALG and BW-CMC bigels were similar, suggesting that the crystal structure of our bigels is not highly dependent on the type of polymer used in the hydrogel.

Through CLSM images, it was observed that BW-ALG and BW-CMC bigels formed a matrix with a hydrogel-in-oleogel arrangement or water-in-oil structure, where the hydrogel phase was incorporated inside the oleogel matrix (Figure 1a–c). On the other hand, the BW-O showed a dendritic network formed by needle crystals, typical for BW oleogels [16,24]. In the images of bigels, the needle-like structure of BW cannot be easily seen as in the BW-O. This fact could suggest that the shear in the bigel preparation process may destroy the more ordered structure of the BW of oleogel or that the BW crystals would dissipate with the aqueous content within the mixture in accordance with what was found by Martins et al. [3] and Fasolin et al. [11] for bigels.

The type of gelling agent had an effect on the binding capacity; using ALG instead, CMC resulted in a higher binding capacity (Table 2). However, the binding capacity of the bigels dropped significantly compared to the BW-O. This means that the oil retention capacity of the bigels was negatively affected by the preparation process. As observed in X-ray diffraction analyses, the crystallinity of BW-ALG and BW-CMC bigels is lower than that of oleogel, suggesting that the homogenization process using high shear rates could destroy the crystal structure formed in the original oleogel, which could cause the resulting bigel matrix to have less oil-retention capacity.

#### 2.1.4. Texture and Rheology

**Figure 2:**
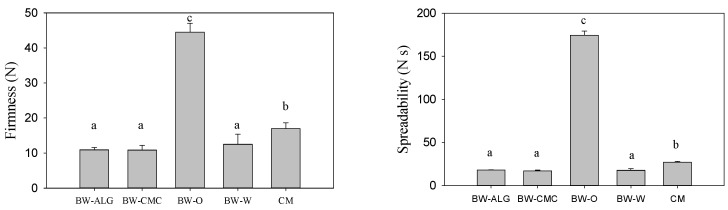

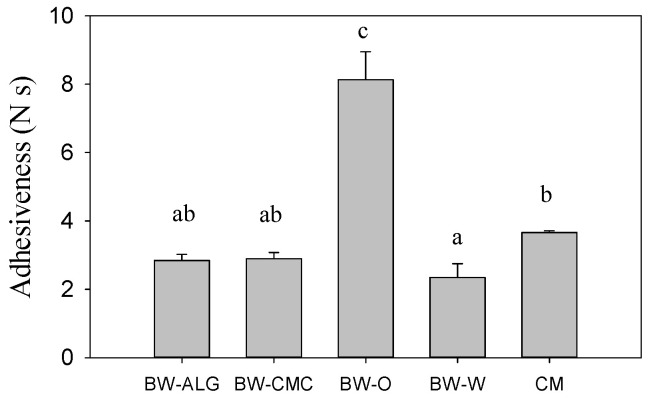
Firmness, spreadability, and adhesiveness of bigels (BW-ALG and BW-CMC), oleogel (BW-O), emulsion of BW-O with water (BW-W), and commercial margarine (CM). Means with different superscript letters in columns are significantly different (*p* ≤ 0.05) for one-way ANOVA and Tukey’s test.

The values of the firmness, spreadability, and adhesiveness of BW-ALG and BW-CMC were similar (Figure 2) and significantly lower than that of BW-O. The BW-W emulsion resulted in firmness, spreadability, and adhesiveness similar to both bigels. Moreover, the textural parameters of bigels were closer to that of commercial margarine (CM). However, the textural analysis indicates that there is no significant effect by using ALG or CMC hydrogels or even water.

Other authors have reported different effects of mixing the two phases of bigels, changing the rheological behavior and mechanical properties of the original matrices, where the bigel components lead to changes in network strength; these changes could be related to interactions between the disperse and continuous phases [11]. For example, in bigels prepared with medium-chain triglycerides Neobee-beeswax (3 and 6%) oleogel and ALG hydrogel (at 2%), mixed at 600 rpm, the oleogel fraction disrupted the alginate network (forming oleogel-in-hydrogel matrix) and consequently decreased the mechanical properties of the bigels compared to the ALG hydrogel; however, the firmness of bigels was higher than that of the original oleogels [3]. In our case, the hydrogel disrupted the oleogel continuous phase, and consequently, the mechanical properties of bigels decreased compared to the oleogel but were improved compared to the hydrogels (data not shown).

A rheological test was performed to characterize the viscoelastic properties of the BW-ALG and BW-CMC bigels and compare them with the rheological properties of BW-O, and hydrogels ALG and CMC at 3%. Additionally, bigels were compared with the BW-W emulsion to determine the effect of hydrogel on the rheological properties of bigels. Moreover, bigels were compared with a CM. 

**Figure 3:**
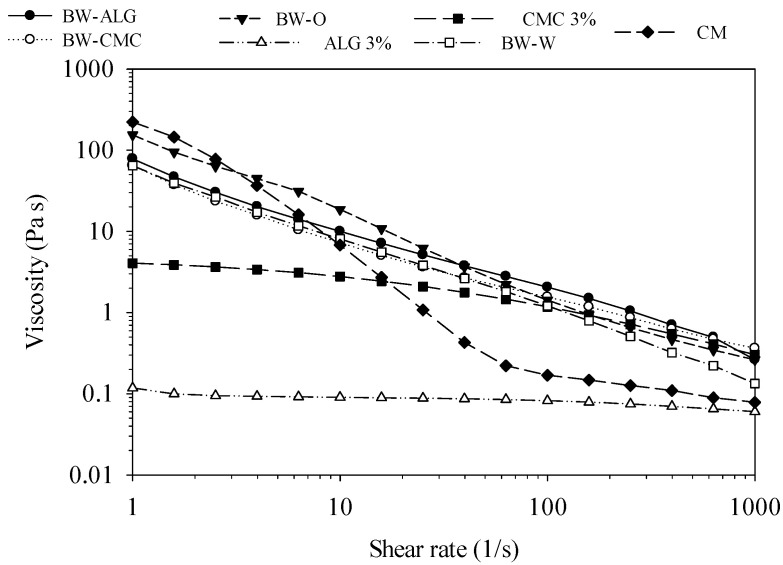
Viscosity as a function of shear rate of bigels (BW-ALG and BW-CMC), oleogel (BW-O), sodium alginate hydrogel (ALG 3%), carboxymethylcellulose hydrogel (CMC 3%), emulsion of BW-O with distilled water (BW-W), and commercial margarine (CM).

The viscosity of all analyzed samples decreased with increasing shear rate, showing non-Newtonian pseudoplastic flow or shear-thinning behavior (Figure 3). The viscosity of the BW-ALG and BW-CMC bigels decreased in a similar way as the shear rate increased, but the viscosity value of BW-ALG was slightly higher than that of BW-CMC (despite ALG having a lower viscosity than CMC). The viscosity of BW-O was higher than that of the bigels at a low shear rate but it decreased faster; thus, from a shear rate of 40 (1/s), it was lower than both bigels. The viscosity of the BW-W emulsion behaved similarly to both bigels, but after a shear rate of 100 (1/s) its value was lower than that of the bigels. This indicates that the presence of hydrogels instead of water in the matrix makes it possible to have a higher apparent viscosity at high shear rates, likely due to intermolecular hydrogen bonding in the polymers forming the hydrogels that can increase the rigidity of the bigel matrices [25]. Pure hydrogels presented different behavior: the CMC hydrogel had a viscosity lower than the bigels and oleogel and decreased slowly as the shear rate increased, being very close to the oleogel after a shear rate of 100 (1/s). The ALG hydrogel had the lowest viscosity, remaining almost constant over shear rate. Finally, the viscosity of CM behaved completely differently to the bigels: at the low shear rate, it presented the highest viscosity, but this decreased faster as the shear rate increased, and after a shear rate of 50 (1/s) its viscosity was low, closer to the viscosity of the ALG (which had the lowest viscosity), indicating that the fat droplets of the emulsion inside the commercial margarine gradually form an optimal orientation along the flow direction. The non-Newtonian pseudoplastic behavior of bigels has been reported before, indicating that this characteristic contributes to spreadability when applied on a surface, meaning that at high shear rates, the bigels can flow readily, while at low shear rates bigels can adopt a higher consistency (higher apparent viscosity) [25].

**Figure 4:**
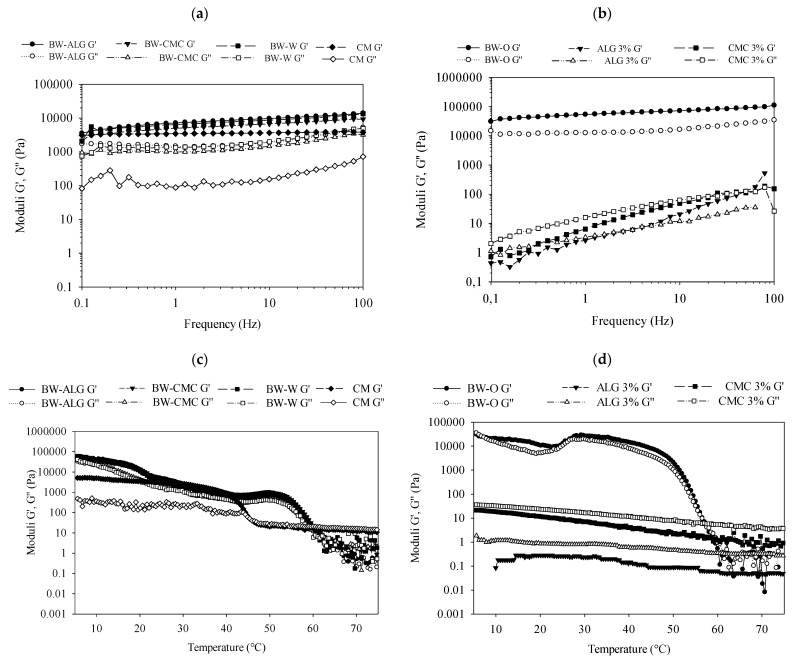
Rheological properties of bigels (BW-ALG and BW-CMC), oleogel (BW-O), sodium alginate hydrogel (ALG 3%), carboxymethylcellulose hydrogel (CMC 3%), emulsion of BW-O with distilled water (BW-W), and commercial margarine (CM): (**a**,**b**) moduli (elastic (G’) and viscous (G″)) during frequency sweep, (**c**,**d**) and moduli during temperature sweep.

A frequency sweep made in the linear viscoelastic region of the bigels showed that the response of the BW-ALG and BW-CMC bigels was largely dominated by the solid component, where G’ was larger than G’’ for the entire frequency range (Figure 4a); moreover, G’ and G″ were slightly increased with the increase in frequency, and there was no cross-over, behavior typical of elastic networks, meaning that the mixture of oleogel and hydrogel produced bigels with stable performance [3]. The rheological response of the BW-O showed that G’ was larger than G″ (Figure 4b), indicating that typical gel networks were formed [3]. An increase of the moduli was observed when the frequency application was amplified, but this remained solid-like for all the frequency ranges (G’ > G″). The elastic modulus of BW-O was larger than that of the bigels, which is consistent with the results of the firmness analysis. For the ALG and CMC hydrogels, G’ was smaller than G″ in a wide range of the studied frequency, specifically at a lower frequency (Figure 4b), exhibiting a liquid-like performance; however, the modulus of ALG after a frequency close to 10 Hz showed a change in its behavior, being G’ > G″. The rheological response of BW-W was similar to that of BW-ALG bigel, with G’ values higher than G″ in the entire frequency range, with the moduli being higher than that of BW-CMC (Figure 4a). In the case of the CM, it can be seen that G’ was higher than G″, indicating solid-like behavior; however, G’ was lower than that of the BW-ALG and BW-CMC bigels, and it remained almost constant as the frequency increased, while G’’ increased as the frequency increased (Figure 4a). These findings showed that bigels exhibited rheological behavior completely different from the CM (which is also a water-in-oil emulsion).

The thermodynamic properties of the bigels, evaluated by the temperature sweep test in the rheometer, showed that from an earlier stage of heating (5 °C), the moduli of the BW-ALG and BW-CMC bigels decreased (Figure 4c). A faster decrease in modulus was observed from approximately 50 °C in the bigels, to 70 and 67 °C for BW-ALG and BW-CMC, respectively; from these temperatures, G’’ was higher than G’, suggesting that the systems had turned into viscous or liquid matrices [10]. The BW-O showed a decrease in moduli from 5 °C. After 47 °C, a faster decrease was observed until 62 °C, suggesting a phase transition of samples occurred in this range (Figure 4d). At temperatures above 62 °C, the oleogels behaved like a liquid. Other authors have reported that the melting temperature of BW oleogels (at 8% BW concentration in medium-chain triglycerides or long-chain triglycerides such as oil phase or sunflower oil) is about 50–63 °C, but this temperature depends on BW concentration; as the oleogelator concentration increases, the melting temperature increases [26,27]. The differences in the critical temperatures of bigels compared to pure oleogel suggest that alginate and CMC interactions could delay the onset destabilization of the system. Likewise, the temperature at which the system had turned into a viscous system shifted from 62 °C for BW-O to 67 °C and 70 °C for BW-CMC and BW-ALG, respectively. These results propose that together with the melting of BW crystals (or the structural breakup of the BW-O precursor), the dissociation of cross-linked CMC and alginate might occur at higher temperatures. The changes could consist of the evaporation of physically bound water and hydrogen-bonding variations [28,29]. Thus, the changes in the melting transition temperatures of bigels compared to the oleogel demonstrate that the hydrogel type has an effect on the thermal stability of bigels. In the case of hydrogels, G’ and G″ decreased almost constantly with the increase of temperature, and no cross-over of modulus was observed in the studied temperature range. For BW-W samples, the modulus decreased over temperature; the faster decrease was observed at 63 °C, a value close to that of the oleogel and lower than that of the bigels. These results suggest that by changing the composition of the aqueous phase of bigels, the phase transition temperature can be modulated.

In the case of the CM, the rheological behavior at temperature sweep was very different from the bigels. A slow decrease in moduli as the temperature increased was found until 40 °C approximately; then, until 46 °C, the fastest decrease rate was observed, suggesting that in this range the phase transition occurs. After 46 °C, G″ was higher than G’, indicating a liquid-like performance. Thus, the CM had a lower phase transition temperature than the BW-ALG and BW-CMC bigels, BW-W, and the BW-O.

### 2.2. Bigels as a Fat Replacer in Cookies

The bigels of BW-ALG and BW-CMC were used as a total fat replacer in the cookie formulation. Cookies made with canola oil and BW-O were compared with cookies made with bigels. Moreover, cookies made with CM were prepared to know the effect of adding a fat matter with textural parameters close to bigels but with different rheological behavior. As a control, cookies were prepared with the original shortening, butter.

The moisture content of cookies made with different fat types varied among samples from 3.08 ± 0.18 to 11.88 ± 0.66% (Table 3). To use bigels as fat matter in cookies, the moisture was close to double the original cookies (containing butter). The BW-O cookies had the lowest moisture content, even lower than the original cookies, whereas using canola oil cookies presented moisture similar to the original cookies. On the other hand, using CM the moisture was the highest, similar to cookies made with the BW-ALG bigel. The highest moisture content was found in cookies that have fat matter with water in their composition; this can be attributed to the amount of water present in the fat matter itself and/or to the higher water retention capacity of such matrices, similar to Yilmaz and Ogutcu [7], who compared cookies with a commercial bakery shortening (containing water) with BW and sunflower oleogels.

The hardness of the cookies made with the bigels (either BW-ALG or BW-CMC) was slightly lower than that of the cookies made with the original shortening (Table 3), although these values were not statistically different among them. With the BW-O, the hardness rose slightly compared to the cookies with original shortening, but this increase was also not statistically significant. However, with canola oil in the cookie formulation, the hardness rose significantly to twice the cookies made with the original shortening. With CM, the hardness of the cookies was halved compared to the cookies made with the original shortening. The fracturability, known as the distance that a cookie is deformed before breaking [30], for cookies made with the bigels was more than three times higher than that of cookies made with the original shortening (Table 3). For cookies made with the BW-O and with canola oil, the fracturability was low, similar to cookies made with the original shortening. With CM, the fracturability was close to three times higher than cookies with the original shortening, similar to cookies with bigels. Fracturability was correlated positively (r = 0.9297) to the moisture content of cookies, meaning that cookies made with bigels with higher moisture had a higher distance at the point of maximum force (less brittle). 

The geometry of the cookies made with bigels does not change the width of the cookies compared with the cookies made with the original shortening, similarly to what was found in the cookies made with BW-O, canola oil, and CM. However, the thickness of cookies depends on the fat matter used. Bigels caused the cookies to be thicker than with the original shortening. With the BW-O, the cookies had a similar thickness to the cookies with the original shortening, but with canola oil, the cookies were thicker than in the cookies with the original shortening, similar to the cookies with bigels. The cookies made with CM presented a thickness similar to the original shortening.

These results indicate that BW-ALG and BW-CMC bigels as fat replacers cause changes to the physical properties of cookies, but the change in the formulation (using ALG or CMC hydrogel) does not affect the magnitude of these changes.

Fat plays an important role in the preparation of cookies. Harder fat molecules form a film around the starch and protein, the particles that conform to the wheat flour; thus, starch molecules have limited access to water, delaying gelatinization and preventing the proteins from interacting with each other to form the gluten network. This results in a weak network of gluten formed by cookie dough, with a porous structure. Moreover, during mixing, plastic fats can trap and retain air. However, when liquid oil is used, it is dispersed throughout the dough in the form of globules (poorly distributed around the flour molecules), being less effective in their roles of shortening (the flour molecules will remain accessible to interact with water) and aeration [14,31]. Therefore, the use of liquid canola oil resulted in the hardest cookies, while the use of oleogels (structured oil) was able to better fulfill the roles of fats in the dough, achieving cookies with similar physical characteristics to the cookies with the original shortening. 

Other studies have reported the use of structured monoglyceride (gel) in an emulsion to replace shortening in cookies, but this matrix cannot mimic the functionality of traditional shortening (i.e., cookies with gel emulsion were harder than cookies with traditional shortening). Although the gel emulsion achieved good functionality in the dough, this was potentially disrupted by heating during the baking process [14], likely due to the use of liquid oil in the structured gel. In our research, the use of bigels, an emulsion formed by two gel phases, does not increase the hardness like other types of emulsions do, suggesting that bigels largely allow fat (structured oil) to satisfy their functional role despite containing less lipid (compared to original shortening and BW-O). 

Some carbohydrate-based fat replacers that form a gel-like matrix with water have been evaluated in cookies and have been reported to imitate fat by binding water and providing lubricity, body, and a pleasing mouth sensation [32,33]. However, other authors pointed out that in general, the cookies prepared with carbohydrate-based fat replacers are harder, more brittle, and have a higher moisture content and water activity than those conventional cookies [34]. In these fat-reduced systems, there is a part of water bound to carbohydrate particles by developing a gel structure. This results in a decrease in the amount of free water, and consequently the cookie dough is hard [11]. For this reason, to obtain a fat reduction without compromising the food quality, a partial substitution of fat is a good option. In our case, despite there being a fat reduction (a reduction in 50% of the lipid content), a compensation could be made by hydrogels that acted as lubricants, allowing no greater development of the gluten network [13], resulting in less hard cookies.

## 3. Conclusions

In the development of bigels, the type of hydrogelator used (CMC or ALG) had an effect on some of their physical and chemical characteristics. In general, bigels maintained the percentage of the fatty acid profile of the BW-O, and the fatty acid profile of canola oil was maintained largely through oleo-gelation with slight differences in the fatty acid amount, evidencing that the process conditions of bigel preparation do not negatively affect the fatty acid profile of the samples; bigels with a high PUFAs content were obtained. The type of gelling agent used to prepare the hydrogel did not influence the fatty acid profile of the bigels. With respect to the lipid oxidation, CMC was used as a gelling agent to obtain bigels with a slightly lower *PV*, although our results indicate that the bigel preparation process did not promote the oxidation of the oil. Moreover, the oxidative stability was not negatively affected by the bigel preparation process, and the use of hydrogels instead of distilled water contributed its improvement. A higher binding capacity was found using ALG as a gelling agent instead of CMC. The resulting bigels were a semi-solid structure with a crystalline and an amorphous component, with a firmness lower than the pure oleogel but higher than the pure hydrogel. The type of polymer used as a gelling agent in bigels (ALG or CMC) did not affect the textural properties of the bigels. The rheological analyses showed that BW-ALG and BW-CMC exhibited shear-thinning behaviors over the entire measured shear rates at 25 °C; additionally, the bigel had a stable performance. Thermodynamic analyses showed that the phase transition temperature of bigels could be modulated by changing their composition; the BW-ALG bigel presented a higher transition temperature than the BW-CMC bigel, and both bigels presented a higher transition temperature than the BW-W and BW-O.

Using bigels as a fat replacer in cookies, the hardness was similar to cookies with the original shortening; however, the fracturability of these cookies was significantly higher. The width of the bigel cookies was not altered, but the thickness and moisture were significantly higher. Using liquid oil resulted in cookies with the highest hardness and thickness. This demonstrated the high potential of the use of bigels as a fat replacer. However, more research is needed to find the conditions to realize bigels that equal all functionalities of fats, for example, modifying the range of study variables; studying other types of gelling agents; and incorporating other variables such as homogenization temperature, the proportion of replacement, and others.

## 4. Materials and Methods

### 4.1. Materials

Cold-pressed canola oil was purchased from Canola de Vida (Osorno, Chile). Beeswax (bleached) and sodium alginate were acquired from Sigma-Aldrich (Santiago, Chile). Carboxymethylcellulose (800–3100 mPas), *p*-anisidine, potassium iodide, and isooctane were acquired from Merck (Santiago, Chile). Glacial acetic acid was purchased from Winkler (Santiago, Chile).

### 4.2. Bigel Preparation

Initially, the oleogel and hydrogel were prepared separately. After that, the two gels were mixed at room temperature to obtain a homogenized bigel.

Oleogels (BW-O) were prepared by mixing the 10% BW with canola oil. The mixture was heated until 70 °C and stirred (300 rpm) for 30 min in total. Then, the resulting solution was immediately transferred to silicone molds and kept at room temperature for at least 2 h. After that, the oleogels were stored in a refrigerator (6 °C).

Hydrogels were prepared by mixing the polymer (ALG or CMC) with distilled water (3% *w*/*w*). The mixture was stirred at room temperature until complete dissolution was achieved (about 24 h). Then, the hydrogels were stored in a refrigerator (6 °C).

Bigels were prepared by mixing the oleogel with the hydrogel in proportions of 50/50 (*w*/*w*) in batches of 35 g. Each mixture was conditioned at 30 °C in an oven (ZRD A5110, Zhicheng, Shanghai, China) for at least 1 h. Then, the oleogel and hydrogel were mixed using a homogenizer (Benchtop, ProScientific, Oxford, CT, USA) at mixing rates of 2500 rpm. Bigels were stored in glass containers in a refrigerator for 24 h before being analyzed. The conditions to prepare the bigels were selected from previous experiments that looked for bigels with solid-like texture (results not shown).

### 4.3. Bigel Characterization

#### 4.3.1. Fatty Acid Composition 

To determine the effect of the process to prepare the bigels in the fatty acid profile, the fatty acid composition of bigels was compared with the canola oil, and the oleogel was used to made these bigels. The fatty acid composition was determined by the AOCS Official Method Ce 1f-96, using gas chromatography (7820A, Agilent Technologies, Santa Clara, CA, USA) equipped with a flame ionization detector. 

#### 4.3.2. Lipid Oxidation 

Lipid oxidation was evaluated in bigels and compared with that of canola oil and BW-O. The peroxide value (*PV*) and *p*-anisidine value (*AV*) were determined to monitor the extent of primary and secondary oxidation, respectively. For *PV* determination, the method based on the AOCS recommended official method Cd 8-53 was used. Briefly, 5 g of the sample was dissolved in a 30 mL mixture of 3:2 acetic acid/chloroform. Then, 0.5 mL of KI saturated solution was added and mixed with 30 mL of distilled water. One ml of starch (1%) was added, and after that, the mixture was titrated with sodium thiosulfate 0.01 N. The *PV* value was calculated as follows [35]:

(1) PV=(S−B)·M·1000/W

where *S* is the amount of sodium thiosulfate in the sample titration in mL, *B* is the amount of sodium thiosulfate in the blank titration in mL, *M* is the molarity of the thiosulfate solution, and *W* is the weight of sample in g.

The *AV* of samples was determined spectrophotometrically using the method of AOCS Cd 18-90 described by Mohanan, Nickerson, and Ghosh [19]. One gram of sample was dissolved in 25 mL of isooctane to obtain the sample solution. On the other hand, 0.25 g of *p*-anisidine was dissolved in 100 mL of glacial acetic acid to obtain the *p*-anisidine solution. One mL of *p*-anisidine solution was added to 5 mL of sample solution and to 5 mL of isooctane (blank). After 10 min, the absorbance of the sample solution, the sample solution with *p*-anisidine solution, and isooctane with the *p*-anisidine solution was measured at 350 nm using the UV–vis spectrophotometer (Synergy HT, BioTek Instruments Inc., Winooski, VT, USA). The *AV* was calculated [19]:

(2) $$AV=25\cdot \frac{1.25\cdot \left(A_{C}-A_{S}\right)}{W}$$

where *A_C_* is the absorbance of isooctane with *p*-anisidine solution, *A_S_* is the absorbance of sample solution less the absorbance of sample solution with *p*-anisidine solution, and *w* is the weight of the sample in grams.

#### 4.3.3. Oxidative Stability

The oxidative stability of samples was determined using the Rancimat test. The Rancimat test is an accelerated technique used for the assessment of the oxidative stability of edible fats and oils [36]. Five grams of the sample was put in the Rancimat (743, Metrohm AG, Herisau, Switzerland) instrument at accelerated oxidation conditions, at 100 °C and 10 L/h of air flow. The Rancimat results are given as a curve of conductivity as a function of time; thus, the induction period was determined from the inflection point of the conductivity curve [36].

#### 4.3.4. X-ray Diffraction

To study the crystalline polymorphism of the bigels, X-ray diffraction analysis was done. The samples were analyzed in an X-ray diffractometer (D8 Advance, Bruker, Karlsruhe, Germany) equipped with a CuK_α1,2_; a K_β_ X-ray tube was set to 40 kV and 40 mA. The samples were mounted on a Lucita sample plate and gently compressed by hand. Scans were collected from 2θ = 2°–50° with step sizes of 0.02° at 1 s per step. The EVA (Bruker Corp., Billerica, MA, USA) software was used to identify and analyze the peaks. 

#### 4.3.5. Confocal Laser Scanning Microscopy (CLSM)

The internal phase distribution in bigels was analyzed by CLSM. Nile Red (30 µM solution in acetone) was used to stain the oil phase of the samples. The samples were exposed to an argon laser (488 nm) at 100 × magnification using a Fluoview 1000 microscope (Olympus, Tokyo, Japan).

#### 4.3.6. Binding Capacity

The centrifuge method was used to determine the oil and/or water loss values of the bigel samples, called binding capacity. One gram of bigel was placed in a Falcon tube and centrifuged at 7000 rpm for 40 min. The excess liquid was decanted onto a paper cloth, and the mass of the Falcon tube with the remaining bigel was weighed. The binding capacity (*BC*) was calculated by the following equation [37,38]:

(3) $$BC=\frac{\left[\left(m_{1}-m\right)-\left(m_{2}-m\right)\right]}{\left(m_{1}-m\right)}\times 100\%$$

where *m* is the mass of empty Falcon tube, *m*_1_ is the mass of the Falcon tube with the initial sample, and *m*_2_ is the mass of the Falcon tube with the final sample.

#### 4.3.7. Texture Determination

For comparison of the BW-ALG and BW-CMC bigels in terms of firmness, the spreadability test was carried out using the TTC spreadability fixture in the texture analyzer TA.XT PlusC (Stable Micro System, Surrey, UK) and exponent connect software version 7.0.6.0 (Stable Micro System, Surrey, UK). The samples were placed in a conical female probe and stored at 6 °C for 24 h. For analysis, the male cone penetrated the sample at a 90° angle with a test speed of 2 mm/s and a distance of 15 mm. The post-test speed was 10 mm/s. The parameters determined were hardness (maximum force, N); spreadability, the difficulty level to spread the margarine (or bigel, or oleogel or emulsion samples) as a thin and uniform layer (positive area, N s); and adhesiveness, the adherence of the product to a surface (negative area, N s) [39]. 

#### 4.3.8. Rheology

Rheological measurements were taken of the oleogel and bigel samples using a rheometer (Discovery HR2, TA Instruments, New Castle, DE, USA), equipped with a flat parallel plate geometry (stainless steel, 50 mm diameter, 1000 µm gap). The samples were placed carefully on the plate and covered with a solvent trap to avoid water evaporation. The TRIOS software package (TA Instruments, New Castle, DE, USA) was used to control the equipment and to acquire rheological parameters. The steady-shear flow measurements were taken at 25 °C in a shear rate range of 1–1000 s^−1^. The range of linear viscosity values for the samples was obtained from the elastic modulus (G’) vs. the oscillatory strain (%) plot. Thus, the viscoelastic behavior of the samples was measured under oscillatory at 1 Hz and from 0.001 to 20%. In the frequency sweep test, the temperature was held at 25 °C, and the response of the moduli (G’, G″, Tan δ) to increasing frequency (0.1 to 100 Hz) at a strain of 0.01% within the linear viscosity region was measured. Finally, temperature sweep tests were performed to evaluate the thermal stability of the oleogels from 5 °C to 90 °C, at a linear heating rate of 5 °C/min, a shear strain of 0.01%, and 1 Hz. Three replicates of each sample were recorded for every test.

### 4.4. Preparation of Cookies

Six different types of cookies were prepared to vary the full-fat matter using (Table 4): BW-ALG bigel, BW-CMC bigel, BW-O, canola oil, commercial margarine (CM) with firmness nearly to that of bigels, and original shortening. Cookies were produced using the following ingredients: wheat flour 500 g, fat 250 g (as buttercream for original shortening, 125 g BW-O + 125 g ALG hydrogel for BW-ALG bigel, 125 g BW-O + 125 g CMC hydrogel for BW-CMC bigel, canola oil, or CM), powdered sugar 230 g, chocolate chips 180 g, NaHCO_3_ 5 g, vanilla aroma 10 mL, and two eggs. The cookies were supplied by Magnolia Cake Shop (Temuco, Chile). Cookies were wrapped in a paper bag and stored in a dry environment until analysis (after 24 h).

### 4.5. Characterization of Cookies

#### 4.5.1. Texture Analysis

The hardness and fracturability of the cookie samples were determined using a texture analyzer (TA.XT Plus, Stable Micro Systems, Godalming, UK). Cookies were compressed (a distance of 10 mm) at a test speed of 2 mm/s, using a 3-point bending attachment. The maximum (or break) force was referred to as hardness, and the distance at the point of maximum force (or break) corresponds to the resistance of the sample to bend and so relates to the fracturability [31]. 

#### 4.5.2. Geometry of Cookies

The thickness and width of cookies were measured using a caliper. The spread ratio of the cookies was calculated by dividing the width by the thickness [31]. Five measurements were taken.

#### 4.5.3. Moisture Content

The moisture of cookies was determined by drying samples using an infrared moisture analyzer (MA37-1, Sartorius, Göttingen, Germany). Results were expressed on a wet weight basis. This analysis was done in duplicate.

### 4.6. Statistical Analysis

To determine whether there were significant differences among the samples, a one-way analysis of variance (ANOVA) was carried out with a significance level set at 0.05. In case of significant differences found with the ANOVA, Tukey’s test was performed using the Statgraphics Centurion XV (version 15.1.02) software.

## Figures and Tables

**Table 1:** Fatty acid profile (g/100 g of sample) of analyzed samples.

Fatty Acid	Canola Oil	BW-O	BW-ALG	BW-CMC
Saturated fatty acids (SFAs)				
C12:0	0.02	0.05	0.03	0.04
C14:0	0.08	0.10	0.05	0.07
C15:0	0.02	0.02	0.01	0.01
C16:0	4.49	6.20	3.25	3.30
C17:0	0.12	0.12	0.06	0.06
C18:0	1.51	1.73	0.92	0.94
C20:0	0.50	0.50	0.26	0.26
C21:0	0.03	0.02	0.00	0.00
C22:0	0.31	0.37	0.19	0.20
C24:0	0.08	0.43	0.24	0.23
Total SFAs	7.16	9.54	5.01	5.11
Monounsaturated fatty acids (MUFAs)				
C14:1	0.00	0.05	0.02	0.03
C15:1	0.01	0.02	0.00	0.01
C16:1	0.22	0.20	0.11	0.12
C18:1	57.76	56.19	28.75	28.21
C 20:1n9	1.14	1.14	0.57	0.57
C 22:1n9	0.21	0.20	0.11	0.10
C 24:1	0.13	0.13	0.07	0.07
Total MUFAs	59.47	57.93	29.63	29.11
Polyunsaturated fatty acid (PUFAs)				
C 18:2n6	18.72	17.30	8.49	8.38
C 18:3n3	10.02	8.83	4.14	4.14
C 20:2n6	0.08	0.08	0.01	0.04
C 20:4n6	0.00	0.00	0.00	0.01
C 20:5n3	0.00	0.03	0.00	0.01
C 22:2	0.02	0.02	0.01	0.01
Total PUFAs	28.84	26.26	12.65	12.59
Trans fatty acids	0.00	0.00	0.00	0.00

**Table 2:** Physical and chemical properties of bigels (BW-ALG and BW-CMC, oleogel (BW-O), the mixture of BW-O with distilled water (BW-W), and canola oil).

Sample	Peroxide Value (meq O_2_/kg Sample)	*p*-Anisidine Value	Induction Time (h)	Binding Capacity (%)
BW-ALG	0.85 ± 0.12 ^b^	2.08 ± 0.10 ^a^	14.1 ± 1.0 ^b^	87.6 ± 0.5 ^c^
BW-CMC	0.45 ± 0.12 ^a^	2.03 ± 0.03 ^a^	15.1 ± 2.3 ^bc^	85.6 ± 0.3 ^b^
BW-O	2.04 ± 0.12 ^c^	2.45 ± 0.14 ^b^	17.5 ± 0.7 ^c^	99.9 ± 0.1 ^d^
BW-W	0.58 ± 0.00 ^a^	2.32 ± 0.10 ^b^	9.2 ± 1.8 ^a^	83.8 ± 0.5 ^a^
Canola oil	1.98 ± 0.00 ^c^	3.35 ± 0.05 ^c^	16.3 ± 0.6 ^bc^	-

Means with different superscript letters in columns are significantly different (*p* ≤ 0.05) for one-way ANOVA and Tukey’s test.

**Table 3:** Textural characteristics, geometry, and moisture content of cookies prepared from different fats: bigels (BW-ALG and BW-CMC), oleogel (BW-O), canola oil, commercial margarine (CM), and original shortening (OS).

Fat Type	Moisture (%)	Hardness (N)	Fracturability (mm)	Width (cm)	Thickness (cm)
BW-ALG	10.32 ± 0.10 ^cd^	29.04 ± 2.59 ^b^	8.9 ± 1.8 ^b^	5.48 ± 0.30 ^a^	2.61 ± 0.02 ^c^
BW-CMC	8.99 ± 0.77 ^c^	24.87 ± 3.96 ^ab^	9.4 ± 0.5 ^b^	5.53 ± 0.13 ^a^	2.42 ± 0.22 ^c^
BW-O	3.08 ± 0.18 ^a^	36.45 ± 4.99 ^b^	2.4 ± 0.1 ^a^	5.63 ± 0.19 ^a^	1.59 ± 0.11 ^a^
Canola oil	4.61 ± 0.02 ^ab^	65.62 ± 13.92 ^c^	3.3 ± 0.7 ^a^	5.39 ± 0.44 ^a^	2.14 ± 0.48 ^bc^
CM	11.88 ± 0.66 ^d^	14.38 ± 1.49 ^a^	8.4 ± 0.9 ^b^	6.47 ± 0.30 ^b^	1.74 ± 0.17 ^ab^
OS	4.78 ± 0.04 ^b^	30.02 ± 6.72 ^b^	2.6 ± 0.2 ^a^	5.64 ± 0.22 ^a^	1.54 ± 0.16 ^a^

Means with different superscript letters in columns are significantly different (*p* ≤ 0.05) for one-way ANOVA and Tukey’s test.

**Table 4:** The recipe formulations used to prepare the cookies.

Ingredient	Cookie Type					
	BW-ALG	BW-CMC	BW-O	CO	CM	OS
Wheat flour (g)	500	500	500	500	500	500
Fat (g)						
BW-ALG bigel	250	-	-	-	-	-
BW-CMC bigel	-	250	-	-	-	-
BW-O oleogel	-	-	250	-	-	-
Canola oil	-	-	-	250	-	-
Commercial margarine	-	-	-	-	250	-
Buttercream	-	-	-	-	-	250
Powdered sugar (g)	230	230	230	230	230	230
Chocolate chips (g)	180	180	180	180	180	180
NaHCO_3_ (g)	5	5	5	5	5	5
Vanilla aroma (mL)	10	10	10	10	10	10
Eggs (units)	2	2	2	2	2	2

Cookie types prepared using different fats: bigels (BW-ALG and BW-CMC), oleogel (BW-O), canola oil (CO), commercial margarine (CM), and original shortening (OS).
